# Quantitative analysis of pupillometry in isolated third nerve palsy

**DOI:** 10.1371/journal.pone.0208259

**Published:** 2018-11-29

**Authors:** Hyeong Min Kim, Hee Kyung Yang, Jeong-Min Hwang

**Affiliations:** Department of Ophthalmology, Seoul National University College of Medicine, Seoul National University Bundang Hospital, Seongnam, Korea; Charite Universitatsmedizin Berlin, GERMANY

## Abstract

**Objectives:**

To objectively assess pupillary involvement according to various etiologies of acquired isolated third nerve palsy using automated pupillometry, and evaluate the efficacy of digital pupillometry in discriminating compressive lesions from microvascular ischemic third nerve palsy.

**Design:**

Retrospective, observational case series

**Methods:**

A total of 171 subjects were included in this study, consisting of 60 subjects with presumed microvascular ischemic third nerve palsy, 51 with non-ischemic third nerve palsy, and 60 controls whose pupillary light responses were measured using a dynamic automated pupillometer. Subjects with non-ischemic third nerve palsy were divided into subgroups according to their etiology; inflammatory and compressive groups including tumor and aneurysm. Pupillometry parameters including minimum and maximum pupil diameters, constriction latency and ratio, maximum and average constriction velocities and dilation velocity were noted. The diagnostic ability of pupillometry parameters for discriminating compressive vs microvascular ischemic third nerve palsy was evaluated. The inter-eye difference of the involved eye and the uninvolved fellow eye was calculated to adjust for individual variability.

**Results:**

Among all parameters, reduced pupillary constriction ratio was the most specific parameter for detecting non-ischemic third nerve palsy, as a large inter-eye difference beyond the normative range of controls was found in 0% of ischemic, 20% of inflammatory and 60% of compressive third nerve palsy. With the diagnostic criteria using inter-eye differences of 1) minimum pupil diameter > 0.45 mm, or 2) pupillary constriction ratio < -7.5% compared to the fellow eye, the sensitivity and specificity for diagnosing compressive third nerve palsy were 95% and 88%, respectively. In the compressive group, positive correlations were found between the degree of external ophthalmoplegia and constriction ratio (r = 0.615, p<0.001), average constriction velocity (r = 0.591, p = 0.001) and maximum constriction velocity (r = 0.582, p = 0.001).

**Conclusions:**

Abnormal pupillary constriction ratio was highly specific for detecting compressive third nerve palsy, although the sensitivity was not high. Digital pupillometry demonstrated relatively good performance for discriminating compressive lesions from microvascular ischemic third nerve palsy.

## Introduction

Acquired isolated third nerve palsy has been investigated for many years, with numerous studies focusing on the diagnosis, causes, and prognosis of different etiologies. It has been widely accepted that pupil size and reactivity are recognized as major clinical factors for distinguishing the different etiologies of third nerve palsy, notably compressive lesions from microvascular nerve infarction.[1] The peripheral location of pupillomotor fibers along the subarachnoid space is considered as a neuroanatomical explanation for pupil-sparing or pupil-involving third nerve pareses.[2] Pupil-sparing third nerve pareses are known to be caused by microvascular ischemia, whereas pupil-involving pareses occur as a result of compressive or infiltrative lesions. Accordingly, pupillary involvement has been considered to be the hallmark for dangerous causes of third nerve palsy.[3, 4] However, pupillary involvement has also been observed in a few cases of microvascular third nerve palsy.[5–7] A recent study by Keane pointed out that in patients with diabetes, 53% showed pupillary involvement.[8] On the contrary, pupil-sparing third nerve palsies have also been reported in compressive lesions.[9, 10] However, none of the previous studies have objectively documented the extent of pupillary involvement according to various etiologies of third nerve palsy.

Despite the strong emphasis placed on pupillary examination in third nerve palsy, in fact, pupillary involvement is subjectively examined by the physician with limited interobserver reliability.[11] Therefore, automated pupillometry may be considered as a potential method to increase the reliability of measuring pupil reactivity. Nowadays, digital pupillometry can be used to obtain objective features and subtle abnormalities of the pupillary light response and help get an accurate diagnosis in various neuro-ophthalmological diseases.[12, 13] To the best of our knowledge, there are currently no published reports quantitatively analyzing pupillary involvement in third nerve palsy. Herein, we objectively measured pupillary responses in acquired isolated third nerve palsy according to various etiologies using a dynamic pupillometry device, and evaluated the efficacy of digital pupillometry in discriminating compressive lesions from microvascular ischemic third nerve palsy.

## Materials and methods

### Patients

The electronic medical records of 186 patients admitted to the Ophthalmology and Neurology Department of Seoul National University Bundang Hospital (SNUBH) between January 2012 and August 2017 diagnosed as third nerve palsy were retrospectively reviewed. The study was approved by the Institutional Review Board of Seoul National University Bundang Hospital and adhered to the tenets of the Declaration of Helsinki. Patient records and information were anonymized and de-identified before access from the authors and the IRB/ethics committee waived the requirement for informed consent.

The diagnosis of third nerve palsy was made by patients’ history, thorough examinations by both ophthalmologists and neurologists, laboratory tests for infectious diseases and inflammatory markers, cerebrospinal fluid (CSF) analysis if necessary, and neuroimaging studies such as brain and orbit magnetic resonance (MR) imaging and MR angiography. Comprehensively, ‘presumed microvascular ischemic’ third nerve palsy was diagnosed when a patient with isolated third nerve palsy was 50-years-old or more with or without vasculopathic risk factors, had no other neurologic abnormality, no history of trauma, negative neuroimaging and normal laboratory studies on infection and inflammation, and most of all, complete resolution of symptoms within 3 to 6 months after onset.

Meanwhile, the pupillary light reflex is known to be affected by age.[14] To match the patients’ age among etiologic groups, patients under the age of 50 were excluded as presumed microvascular ischemic third nerve palsy invariably occurs in older age.

Exclusion criteria included the following: multiple cranial nerve palsies, third nerve palsy occurring under 50 years of age, other ocular diseases, drugs, congenital/developmental diseases and midbrain lesions that may affect pupillary light responses, previously diagnosed palsy due to other neurologic diseases, insufficient documentation of ocular examination, a history of ocular trauma, and those who did not perform automated pupillometry on initial examination.

We selected age-matched controls from individuals with no definite neuro-ophthalmological diseases and no underlying medical and ocular diseases. Subjects in the control group were chosen from those regularly visiting the ophthalmology department for routine eye examinations and who also underwent digital pupillometry.

### Clinical characteristics

Data included patients’ age, sex, laterality, associated neurologic signs and symptoms at presentation, general conditions, medication, degree of extraocular muscle dysfunction and ptosis on initial and follow-up examinations, and the time to recovery of external ophthalmoplegia. The degree of external ophthalmoplegia was defined as the sum of the four gradings on limitations in duction of the superior rectus, medial rectus, inferior rectus, and inferior oblique muscles. Gradings were marked on a scale from -1 to -4.

We also investigated the etiology of third nerve palsy to compare the clinical findings and pupillometry measurements among groups. Among non-ischemic third nerve palsy, the clinical characteristics of inflammatory third nerve palsy are known to be quite different from those of compressive lesions. Notably, an acute or subacute onset, accompanying pain and rapid response to steroids are exclusive features of inflammation.[15–22] Therefore, patients with non-ischemic third nerve palsy were divided into two subgroups: inflammatory and compressive (tumor and aneurysm) groups.

### Automated pupillometry

Pupillary light reflex (PLR) was obtained and recorded using the PLR-200 pupillometer (NeurOptics Inc., Irvine, USA). PLR-200 pupillometer is an automated monocular infrared pupillometer that records pupil images of each eye separately. PLR of each subject were measured in a consistent order of right eye followed by the left eye. Pupillometry was performed after 3 minutes of dark adaption. Patients were instructed to fixate on a small target object such as a dim flash light at least 3 meters away with the contralateral eye. PLR-200 pupillometer has an eyecup designed for fitting the periorbital area which helps reduce the possibility of light entering the tested eye and standardize stimulus distance and intensity. Stimuli consisted of pulses of light with a fixed intensity of 180 microwatts/cm^2^ and duration of 185 milliseconds. Pupil size measurements were sampled at a frequency of 32 frames per second and lasted up to 5 seconds, allowing a full or partial recovery of the pupil size after light constriction. PLR of each eye was measured twice and the average of data was used. The device has been specifically designed to minimize possible inter-observer variability in the pupillary evaluation.[23]

The seven PLR parameters were presented with pupil response curves: maximum pupil diameter (mm), minimum pupil diameter (mm), pupillary constriction ratio (%), latency (sec), average constriction velocity (ACV, mm/sec), average dilation velocity (ADV, mm/sec), and maximum constriction velocity (MCV, mm/sec).[24–26] Pupillometry was examined at the initial and follow-up examinations, until no further clinical improvement was observed. We also analyzed the effect of vasculopathic risk factors on pupil size, as it has been acknowledged that pupil size can be affected by diabetes.[27–29]

To adjust for individual variability of pupil measurements,[30, 31] the inter-eye difference between the involved eye and the noninvolved fellow eye was analyzed for each parameter in unilateral third nerve palsy. Four cases of bilateral third nerve palsy in the non-ischemic group were excluded in this analysis.

To define subtle pupillary abnormalities in subjects with third nerve palsy, a normative range of inter-eye differences was set for each parameter, which was determined as the extent between minimum and maximum differences between fellow eyes of the control group. The diagnostic ability of each parameter for discriminating compressive optic neuropathy from presumed microvascular third nerve palsy was evaluated.

### Statistical analysis

All data were analyzed using SPSS ver. 21.0 software (IBM Corporation, Armonk, NY, USA). Subgroup comparison of third nerve palsies were performed using Fisher’s exact test and *χ*^2^ test for dichotomous variables, Pearson’s *χ*^2^ test for polytomous variables, one-way ANOVA for continuous parametric variables, and Kruskal-Wallis test for continuous nonparametric variable. One-way ANOVA with Bonferroni adjustment was performed; the results were considered statistically significant when the P-value was less than 0.00625 (0.05/8 Bonferroni adjustment). Otherwise, a P-value of less than 0.05 was considered statistically significant. All data are presented as mean ± standard deviation. The diagnostic efficacy of PLR parameters in discriminating compressive third nerve palsy from microvascular ischemic third nerve palsy was assessed using the area under the receiver operating characteristic curve (AUC). Simple bivariate correlation analysis was conducted to evaluate the relationship between the degree of external ophthalmoplegia and pupil involvement. Subgroup analysis was performed according to complete or incomplete palsy. Complete third nerve palsy was defined as having complete external ophthalmoplegia.

## Results

### Patient characteristics

A total of 111 patients with acquired isolated third nerve palsy who met the inclusion criteria were included for analysis. Sixty patients were diagnosed with presumed ischemic third nerve palsy and 51 patients with non-ischemic third nerve palsy. Among the 51 patients with non-ischemic third nerve palsy, inflammation was the cause in 21 patients, tumor compression in 15 patients, and aneurysmal compression in 15 patients.

Table 1 shows the demographics and clinical characteristics of patients with acquired isolated third nerve palsy, according to their etiologic classification. There was no statistical significance in the mean age of onset and sex among the groups. Vasculopathic risk factors were defined according to the criteria used by Jacobson [17,18]. Diabetes mellitus, hypertension, hyperlipidemia, and prior stroke history were all significantly more frequent in the ischemic group than in the non-ischemic group and controls, as shown in bold (Table 1).

**Table 1:** Demographics and clinical characteristics.

Characteristics		Ischemic	Non-ischemic		Control	*P-*value
			Inflammatory	Compressive		
Number of subjects		60	21	30	60	
Mean onset age		67.3±9.3	64.5±9.6	64.9±9.7	65.0±9.2	0.299*
Male : Female		36 : 24	13 : 8	17 : 13	38 : 22	0.653^†^
Vascular Risk Factors	DM	36 (60%)	9 (18%)		0	**<0.001**^†^
	HTN	43 (72%)	21 (41%)		0	**<0.001**^†^
	HL	30 (50%)	4 (8%)		0	**<0.001**^†^
	Stroke	8 (13%)	1 (2%)		0	**0.032**^†^
Recovery^#^		42 (100%)	18 (100%)	17 (63%)		
Mean recovery time (months)		2.2±0.8	1.7±0.9	3.2±2.2		**0.029**^‡^

DM = Diabetes mellitus; HTN = Hypertension; HL = Hyperlipidemia; Stroke = Previous stroke history

* One-way ANOVA for continuous parametric variables.

^†^ Fisher’s exact test/*χ*^2^ test for dichotomous variables and Pearson’s *χ*^2^ test for polytomous variables.

^‡^ Kruskal-Wallis test for continuous nonparametric variables.

^#^ Follow-up data were available in 42 ischemic, 18 inflammatory, 27 compressive subjects.

*P-*values in boldface indicate statistical significance.

The mean time to maximum recovery of third nerve palsy was measured in patients who showed clinical improvement or external ophthalmoplegia during follow-up examinations. Forty-two out of 60 patients in the ischemic group and 18 out of 21 patients in the inflammatory group had regular follow-up examinations, and all patients showed full recovery from third nerve palsy. In the compressive group, 27 out of 30 patients had follow-up examinations, of which, only 17 patients (63%) showed full or partial recovery of third nerve palsy. The remaining 10 patients (37%) did not show any clinical improvement during the follow-up period of 13.1±3.2 months (range, 9–18). The mean time to recovery were 2.2±0.8 months (range, 1–3) in the ischemic group, 1.7±0.9 months (range, 1–4) in the inflammatory group, and 3.2±2.2 months (range, 1–8) in the compressive group (p = 0.029). Fastest recovery was observed in the inflammatory group, while the slowest recovery was found in the compressive group.

### Quantitative pupillometry in third nerve palsy

Pupillometry findings of the involved eye at the time of initial examination are summarized in Table 2. The maximum and minimum pupil sizes were smaller in the ischemic group than in the non-ischemic and control groups. There was no significant difference in the pupil size among other groups, except for the minimum pupil size, which was slightly larger in the compressive group compared with controls.

**Table 2:** Quantitative analysis of pupillometry in acquired isolated third nerve palsy at the initial examination.

Characteristics	Ischemic (A)	Non-ischemic		Control (D)	*P-*value	A vs B	A vs C	A vs D	B vs C	B vs D	C vs D
		Inflammatory (B)	Compressive (C)								
Maximum pupil size (mm)	4.40±0.82 (2.80, 6.10)	5.35±0.77 (3.20, 6.50)	5.12±1.07 (2.90, 7.10)	5.34±0.82 (3.70, 7.80)	**<0.001**†	**<0.001**	**0.002**	**<0.001**	0.523	1.000	0.298
Minimum pupil size (mm)	3.15±0.68 (2.00, 4.70)	4.20±0.93 (2.20, 5.80)	4.58±1.21 (2.30, 7.10)	3.70±0.69 (2.50, 5.90)	**0.002**†	**<0.001**	**<0.001**	**0.004**	0.294	0.135	**<0.001**
Constriction (%)	28.7±6.4 (14.0, 40.0)	22.4±8.7 (3.0, 36.0)	11.1±10.4 (0.0, 29.0)	30.9±4.2 (24.0,41.0)	**<0.001**‡	**0.007**	**<0.001**	0.355	**<0.001**	**<0.001**	**<0.001**
Latency (sec)	0.26±0.04 (0.19, 0.34)	0.26±0.03 (0.22, 0.34)	0.29±0.24 (0.00, 1.20)	0.23±0.02 (0.19, 0.28)	0.721‡	0.999	0.535	0.622	0.460	0.886	0.082
Average constriction velocity (mm/sec)	2.76±0.70 (1.33, 4.14)	2.63±0.94 (0.16, 3.94)	1.25±1.16 (0.00, 3.44)	3.54±0.48 (2.12, 4.97)	**<0.001**‡	0.922	**<0.001**	**<0.001**	**<0.001**	**<0.001**	**<0.001**
Maximum constriction velocity (mm/sec)	3.73±1.00 (1.85, 5.66)	3.51±1.11 (1.16, 5.18)	1.76±1.45 (0.00, 4.29)	4.65±0.65 (2.77, 6.33)	**<0.001**‡	0.857	**<0.001**	**<0.001**	**<0.001**	**<0.001**	**<0.001**
Average dilation velocity (mm/sec)	0.73±0.23 (0.36, 1.46)	0.59±0.28 (0.01, 1.06)	0.39±0.34 (-0.15, 1.08)	0.88±0.16 (0.50, 1.26)	**<0.001**‡	0.117	**<0.001**	**0.006**	**0.044**	**<0.001**	**<0.001**

† One-way ANOVA for continuous parametric variables.

‡ Kruskal-Wallis test and Mann-Whitney U test for continuous nonparametric variables.

*P*-values in boldface indicate statistical significance.

We analyzed the effect of vascular risk factors on pupil size, as pupil size may be affected by diabetes. [21,22] As a result, in the ischemic group, patients with diabetes mellitus had smaller maximum and minimum pupil sizes compared with non-diabetic patients (p = 0.033,0.022) and controls (all, p<0.001). The noninvolved fellow eye in diabetics also showed a smaller pupil size compared with non-diabetics and controls. However, hypertension, hyperlipidemia, and prior stroke history did not affect pupil size or pupillary light response parameters in all groups.

The pupillary constriction ratio was significantly different among all groups (control = ischemic>inflammatory>compressive), except between the ischemic group and the control group (p = 0.355). Constriction velocities (ACV, MCV) and dilation velocity (ADV) were significantly different among all groups (control > ischemic = inflammatory > compressive), except between the ischemic and inflammatory groups (Table 2).

**Fig 1:**
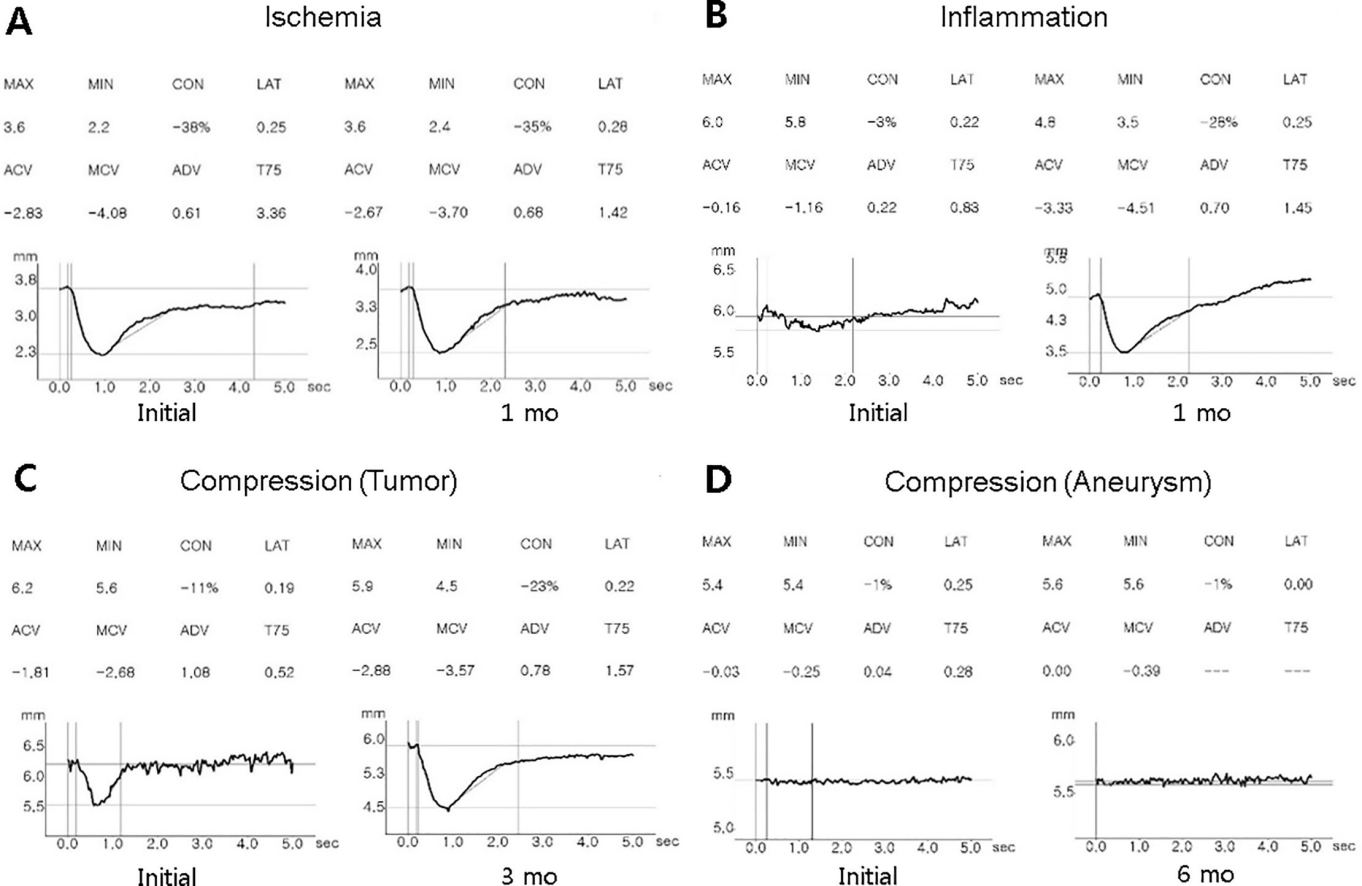
Pupillometry of representative patients in each group at initial (left) and follow-up examinations (right) (A: ischemic, B: inflammatory, C-D: compressive groups). (A) Presumed ischemic third nerve palsy showing normal pupillary light responses. (B) Inflammatory third nerve palsy caused by Tolosa-Hunt syndrome with eye pain, headache, dilated pupil, complete ptosis and ophthalmoplegia. Automated pupillometry shows decreased CON, ACV, MCV and ADV at onset. After 1 month with steroid treatment, pupillary light responses had fully recovered. (C) Compressive third nerve palsy due to parasellar meningioma showing decreased constriction of the pupillary light response. Automated pupillometry shows decreased CON, ACV, and MCV. Three months after stereotactic radiotherapy, full recovery of ptosis, ophthalmoplegia, and pupillary responses were observed. (D) Compressive third nerve palsy due to posterior communicating artery aneurysm with absent pupillary light response on automated pupillometry. After 6 months, no improvement of ptosis, ophthalmoplegia and pupillary light responses were found. ACV = Average constriction velocity; ADV = Average dilation velocity; CON = pupillary constriction ratio (%); MCV = Mean constriction velocity.

Fig 1 shows the representative pupillometer findings in accordance with the etiology of third nerve palsy.

Adjusted pupillometer findings compared with the fellow eye in unilateral cases are described in Table 3. Overall, there were significant differences between the compressive group and other groups in minimum pupil size, constriction ratio, ACV, MCV and ADV. The ischemic, inflammatory, and control groups showed no statistically significant difference with respect to all seven PLR parameters. In addition, there were no significant differences among groups according to the presence or absence of vasculopathic risk factors mentioned above.

**Table 3:** Quantitative analysis of fellow eye-adjusted* pupillometry in acquired isolated third nerve palsy at the initial examination.

Characteristics	Ischemic (A)	Non-ischemic		Control (D)	*P*-value	A vs B	A vs C	A vs D	B vs C	B vs D	C vs D
		Inflammatory (B)	Compressive (C)								
Maximum pupil size (mm)	-0.11±0.48 (-1.90, 1.00)	-0.03±0.50 (-1.30, 0.80)	-0.05±0.74 (-1.60, 1.80)	-0.14±1.06 (-2.10, 2.20)	<0.493^†^	0.981	0.718	0.995	1.000	0.949	0.912
Minimum pupil size (mm)	0.02±0.46 (-1.50, 1.20)	0.24±0.62 (-0.50, 1.80)	0.96±0.80 (-0.30, 2.40)	0.03±0.84 (-1.80, 1.80)	**<0.001**^†^	0.615	**<0.001**	1.000	**<0.001**	0.653	**<0.001**
Constriction (%)	-2.0±5.7 (-13.0, 15.0)	-4.5±8.3 (-20.0, 6.0)	-17.7±12.6 (-42.0, 3.0)	-2.2±5.0 (-13.0, 9.0)	**<0.001**^‡^	0.617	**<0.001**	0.999	**<0.001**	0.688	**<0.001**
Latency (sec)	0.01±0.03 (-0.03, 0.09)	0.01±0.03 (-0.03, 0.06)	0.05±0.23 (-0.28, 0.98)	0.01±0.02 (-0.06, 0.09)	0.464^‡^	0.999	0.242	0.996	0.452	0.992	0.157
Average constriction velocity (mm/sec)	-0.22±0.59 (-1.71, 1.60)	-0.37±1.01 (-2.82, 1.39)	-1.82±1.43 (-4.57, 0.59)	-0.22±0.61 (-1.56, 1.43)	**<0.001**^‡^	0.897	**<0.001**	0.903	**<0.001**	0.914	**<0.001**
Maximum constriction velocity (mm/sec)	-0.24±0.85 (-2.23, 1.60)	-0.49±1.24 (-3.15, 1.63)	-2.21±1.86 (-5.76, 0.98)	-0.26±0.88 (-2.33, 2.35)	**<0.001**^‡^	0.842	**<0.001**	1.000	**<0.001**	0.883	**<0.001**
Average dilation velocity (mm/sec)	-0.03±0.24 (-0.61, 0.64)	-0.16±0.25 (-0.74, 0.40)	-0.39±0.41 (-1.09, 0.25)	-0.05±0.23 (-0.52, 0.48)	**0.001**^‡^	0.326	**0.001**	0.985	0.117	0.491	**0.001**

* The difference between the involved eye and the uninvolved fellow eye to adjust for individual variability.

† One-way ANOVA for continuous parametric variables.

‡ Kruskal-Wallis test and Mann-Whitney U test for continuous nonparametric variables.

P-values in boldface indicate statistical significance.

### Diagnostic ability of quantitative pupillometry for discriminating compressive vs ischemic third nerve palsy

Standard criteria of abnormality for PLR parameters are not established. Therefore, a normative range for each parameter was determined as the extent between minimum and maximum differences between the fellow eyes in the control group. The percentages of subjects outside the normative range of inter-eye difference for each parameter in ischemic, inflammatory and compressive third nerve palsy were as follows; 0%, 20%, 60% for pupillary constriction ratio; 2%, 10%, 50% for ACV, and 0%, 10%, 40% for MCV, respectively. Abnormally reduced pupillary constriction ratio compared to the fellow eye was highly specific for non-ischemic third nerve palsy, as none of the patients with microvascular ischemic third nerve palsy showed abnormal values outside the normative range.

The diagnostic ability of PLR parameters for discriminating compressive third nerve palsy from microvascular ischemic third nerve palsy are summarized in Table 4. With the diagnostic criteria fulfilling at least one of the following: 1) inter-eye difference of minimum pupil diameter _(involved eye–fellow eye)_ > 0.45 mm, 2) inter-eye difference of pupillary constriction ratio _(involved eye–fellow eye)_ < -7.5%, the sensitivity and specificity for diagnosing compressive third nerve palsy were 95% and 88%, respectively.

**Table 4:** Diagnostic ability of automated pupillometry parameters to discriminate compressive vs presumed microvascular ischemic third nerve palsy.

	Cut off value	AUC	Sensitivity (%)	Specificity (%)	Sensitivity at 95% specificity (%)	Specificity at 95% sensitivity (%)
**Inter-eye difference**						
Minimum pupil diameter (mm)	0.45	0.840	83%	65%	17%	50%
CON (%)	-7.5%	0.844	89%	75%	17%	65%
ACV (mm/s)	-0.90	0.825	85%	68%	10%	60%
MCV (mm/s)	-1.12	0.811	88%	68%	17%	55%

AUC = Area under the curve; ACV = Average constriction velocity; CON = Pupillary constriction ratio (%); MCV = Mean constriction velocity; Parameters with an AUC of > 0.700 are presented.

### Correlations of ophthalmoplegia with quantitative pupillometry

There were no patients with complete third nerve palsy in the ischemic and inflammatory groups, but 13 patients in the compressive group were classified as complete palsy. Therefore, we further separated the compressive group into two subgroups for better comparison: complete (13 patients) and incomplete palsy (17 patients). The pupillary constriction ratio, ACV, and MCV were significantly reduced in patients with complete palsy compared with those with incomplete palsy (p = 0.002, 0.004, 0.005, respectively).

Correlations between the degree of external ophthalmoplegia and pupillary involvement were determined for each parameter. There was no significant correlation found in all pupillometry parameters in the ischemic and inflammatory groups; however, in the compressive group, the degree of external ophthalmoplegia was positively correlated with the constriction ratio (r = 0.615, p<0.001), ACV (r = 0.591, p = 0.001), and MCV (r = 0.582, p = 0.001).

In subjects who showed clinical improvement of third nerve palsy, we evaluated the change in pupillometer findings between the initial and final examinations. In the ischemic group and inflammatory group, there were no significant changes in all PLR parameters between the initial and final examination. By contrast, in the compressive group, minimum pupil size showed significant reduction after recovery (p = 0.018). No other pupillometry parameter showed significant change after clinical improvement.

## Discussion

To our knowledge, this study provided the first evidence of automated pupillometry results in third nerve palsy regarding various etiologies of ischemic, inflammatory and compressive lesions. In this study, reduced pupillary constriction ratio compared to the fellow eye was highly specific for non- ischemic third nerve palsy as none of the patients with presumed microvascular ischemic third nerve palsy showed abnormal values outside the normative range. This is contrary to the previous reports that relied on subjective evaluation of pupillary responses.

Interestingly, patients with presumed ischemic third nerve palsy had a smaller pupil size, decreased pupillary constriction velocities (ACV, MCV) and dilation velocity (ADV) compared with controls. After adjusting for confounders, we found that this was related to the presence of diabetes. Small pupils in diabetic patients are known to be caused by diabetic autonomic neuropathy.[27, 28] As the symmetry of pupillary light response in both eyes is relatively preserved, inter-eye comparison is recommended to adjust for various confounders that may affect the pupillary light response such as diabetes.

There were numerous studies and reports analyzing the incidence and etiologies of acquired third nerve palsy. The Mayo Clinic studies included 1491 patients with third nerve palsy and 76% were isolated cases.[3, 4] Among all patients, the cause for the paralysis was diagnosed in only ten of the 127 patients who could be traced and 17% were diagnosed as presumed microvascular ischemic third nerve palsy, while 16% had tumor, 15% occurred after trauma, and 3% were due to aneurysm. [4] The latest study from the University of Southern California Medical Center examined 1400 cases of third nerve palsies, including 500 isolated cases.[8] This study reported that 26% were due to presumed microvascular ischemia with diabetes, while 21% occurred after trauma, 21% due to aneurysm, and 6% had tumor. Complete pupillary sparing was observed in 47% of presumed microvascular ischemic palsy related to diabetes, 35% of Guillain-Barré syndrome, 25% of Miller Fisher syndrome, 9% of tumor, 9% of trauma, and 2% of aneurysm.[8] Another study from the Mayo Clinic identified 145 cases of acquired third nerve palsy over a 37-year period, and the causes were presumed microvascular ischemia (42%), trauma (12%), tumor (11%), post-neurosurgery (10%), and aneurysm (6%).[32] This study reported that pupillary involvement was found in 17% of presumed microvascular ischemic palsy and 64% with compressive third nerve palsy.[32] According to our study, abnormal pupillary light reflex detected by digital pupillometry was highly specific for non-ischemic third nerve palsy, although the sensitivity was low. Previous studies and reports also suggested that anisocoria alone cannot definitively differentiate ischemic causes from compressive third nerve palsies, since compressive lesions, such as tumor and aneurysm, can present without pupil involvement. [9,10]

Nevertheless, previous studies evaluating pupillary involvement in acquired third nerve palsies were solely based on subjective evaluation.[1–10, 15–17, 27, 28, 32]. Subjective determination of mild anisocoria or pupil reactivity may not be accurate as compared to digital pupillometry. Nowadays, since we can measure the pupil size and its motor function more accurately using digital pupillometry, we objectively demonstrated the absence or presence, and extent of pupillary involvement. In our study, an abnormal pupillary constriction ratio outside the normative range of controls determined by automated pupillometry was found in none of the patients with ischemic third nerve palsy. This is contrary to previous reports showing pupillary involvement in 17–53% of presumed microvascular ischemic palsy, particular in patients with diabetes.[5–8] Pupillary involvement in diabetes-associated third nerve palsy is recognized, but the degree of anisocoria is reported to be typically mild, usually less than 1mm.[27, 28, 32] Manual assessment of pupil reactivity is more challenging in small pupils. Therefore, there is a possibility that subjective pupillary assessment in small pupils of diabetic patients may be uncertain. As none of the patients with presumed microvascular ischemic third nerve palsy showed an abnormal pupillary light response by automated pupillometry in our study, pupillary involvement in third nerve palsy may be overestimated in previous reports due to the subjective nature of assessment. A previous report also showed that only 33% of pupils scored as non-reactive by practitioners were actually non-reactive by pupillometry, suggesting that interobserver reliability for subjective pupillary assessment is relatively low.[11]

In the inflammatory group, abnormal PLR parameters measured by automated pupillometry was found in 6 patients (30%). This was higher compared to subjective assessment, as only 2 patients (10%) with Tolosa-Hunt syndrome were initially documented to have pupillary involvement by manual observation. The other 2 patients with decreased constriction ratio were thought to have normal pupillary responses according to the primary care physician’s clinical examination. Previous studies, including case reports, have described the possibility of pupillary involvement in Tolosa-Hunt syndrome.[18, 19, 22, 33] Hung et al.[21] presented that among 25 cases of Tolosa-Hunt syndrome, 6 cases (22%) showed limited pupillary responses. Our results show that pupillary constriction ratio, ACV, and MCV are useful for detecting pupillary involvement in inflammatory third nerve palsies, suggesting the necessity of quantitative pupillometry in the evaluation of pupillary responses in addition to a thorough ocular examination.[34–36]

Positive correlations were found between the degree of external ophthalmoplegia and pupillometry parameters of constriction ratio, average constriction velocity and maximum constriction velocity, only in the compressive group. Therefore, as well as the absence or presence of subtle pupillary involvement, automated pupillometry can help determine the degree of pupillary abnormality and course of recovery. Conversely, total external ophthalmoplegia with disproportionate pupil sparing confirmed by automated pupillometry is almost always caused by microvascular ischemia and may not require urgent neuroimaging.

This study is retrospective and there was not enough number of patients within the compressive group for a meaningful comparison between different etiologies. We excluded 6 patients with midbrain stroke, to exclude central lesions that might interrupt pupillary light responses in both eyes. Further studies with a larger number of subjects are necessary to better analyze the pupillometry data among different etiologies of acquired third nerve palsies. Furthermore, all subjects are Korean, and therefore, the results may not be generalizable to other populations. Lastly, as a major proportion of compressive and inflammatory cases do not show pupil involvement, the sensitivity of dynamic pupillometry was low for predicting non-microvascular etiologies of third nerve palsy. Despite these limitations, our study implies that quantitative pupillometry may be a reliable and sensitive tool for diagnosing pupillary involvement in third nerve palsy.

In conclusion, reduced pupillary constriction ratio compared to the fellow eye detected by digital pupillometry was highly specific for non-ischemic third nerve palsy. Digital pupillometry showed relatively good performance for discriminating compressive lesions from microvascular ischemic third nerve palsy.
